# Bimekizumab-induced paradoxical palmoplantar pustular psoriasis

**DOI:** 10.1016/j.jdcr.2025.12.026

**Published:** 2025-12-24

**Authors:** Ali İmran Özatak, İrem Ertekin, Ramazan Aksoy, Kübra Şimşek, Cumhur İbrahim Başsorgun, Erkan Alpsoy

**Affiliations:** a Department of Dermatology, Akdeniz University Hospital, Antalya, Turkey; b Department of Pathology, Akdeniz University Hospital, Antalya, Turkey

**Keywords:** bimekizumab, cytokine shift, IL-17 inhibitors, palmoplantar pustular psoriasis, paradoxical psoriasis

## Introduction

Psoriasis vulgaris is a chronic, immune-mediated inflammatory skin disease affecting 2% to 3% of the population.^1^ Biological therapies are now widely used to treat moderate-to-severe cases of the disease. Paradoxical reactions (PR) are defined as the emergence of an immune-mediated disease that typically responds to targeted biologic drugs during treatment with this class of drugs.^2^ While biological therapy-related palmoplantar pustular psoriasis (PPP) has been most often linked to tumor necrosis factor (TNF)-α inhibitors, it has also been rarely reported with interleukin 17 (IL-17)A inhibitors.^3^^,^^4^ Bimekizumab is a monoclonal antibody that targets both IL-17A and IL-17F.^5^ To date, cases of paradoxical psoriasis associated with bimekizumab have been reported extremely rarely. This report describes a case of PPP that developed in a patient who had previously been diagnosed with plaque psoriasis and started bimekizumab treatment.

## Case report

**Fig 1:**
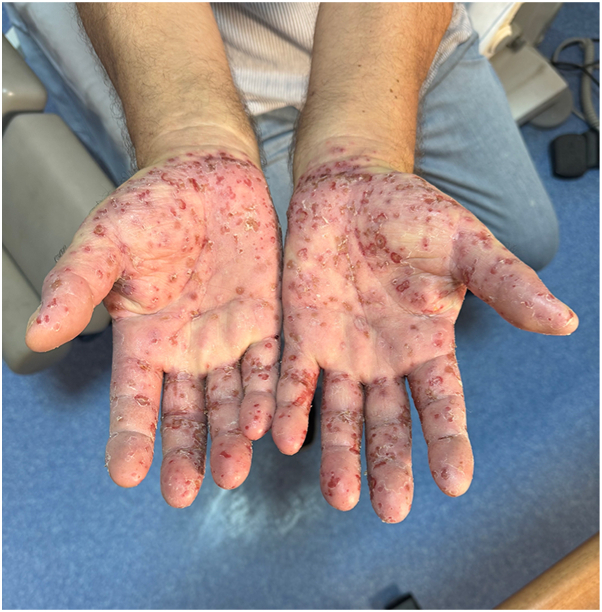
Palmoplantar pustulosis presenting with erosions, desquamation, and pustules on the patient's palms after treatment with bimekizumab for plaque psoriasis.

**Fig 2:**
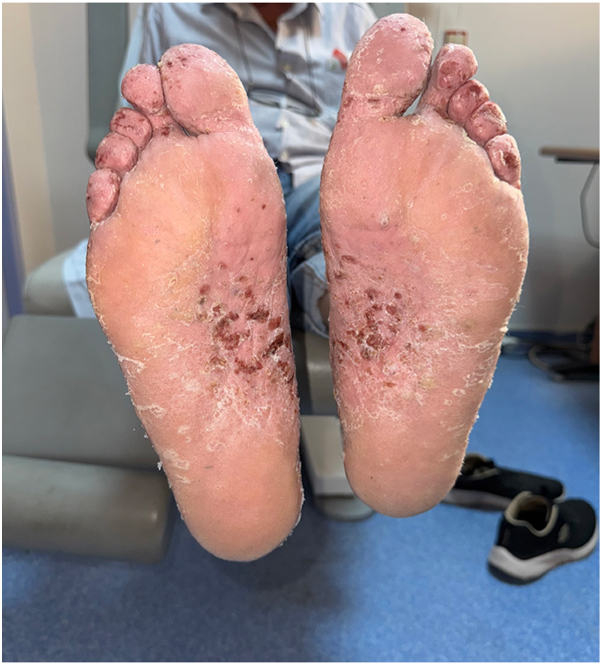
Palmoplantar pustulosis presenting with erosions, desquamation, and pustules on the patient's soles after treatment with bimekizumab for plaque psoriasis.

**Fig 3:**
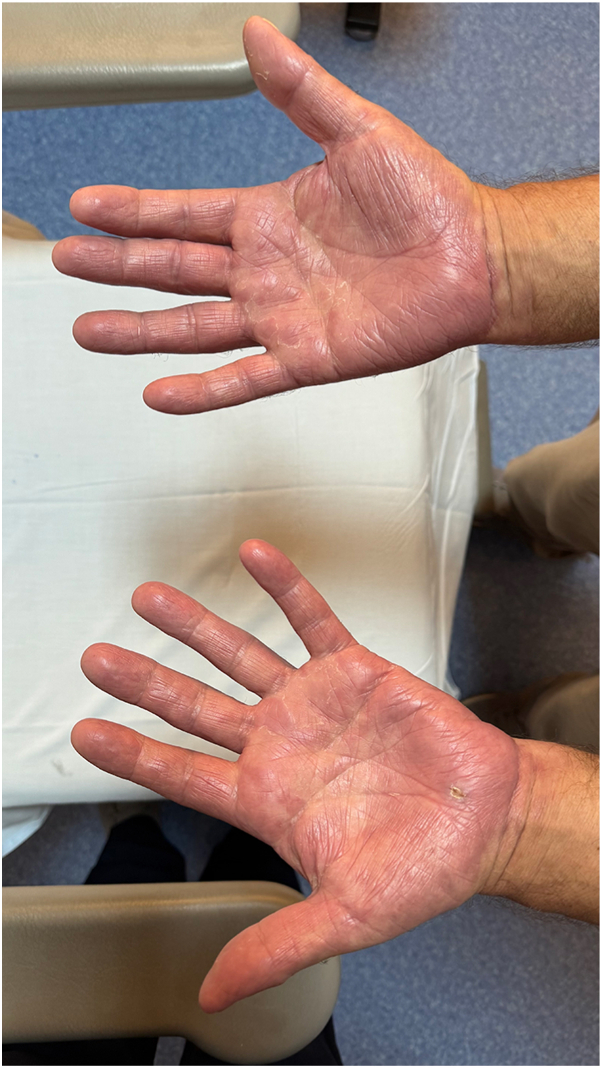
Improvement in the patient's palmoplantar pustulosis, with diminished erythema, scaling, and pustules following discontinuation of bimekizumab and 14 days of treatment with acitretin and topical betamethasone valerate/fusidic acid.

**Fig 4:**
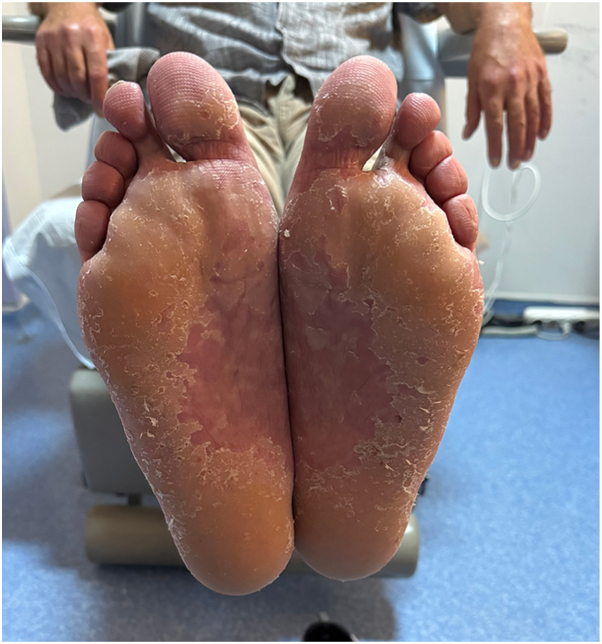
Improvement in the patient's palmoplantar pustulosis, with diminished erythema, crusting, and pustules following discontinuation of bimekizumab and 14 days of treatment with acitretin and topical betamethasone valerate/fusidic acid.

**Fig 5:**
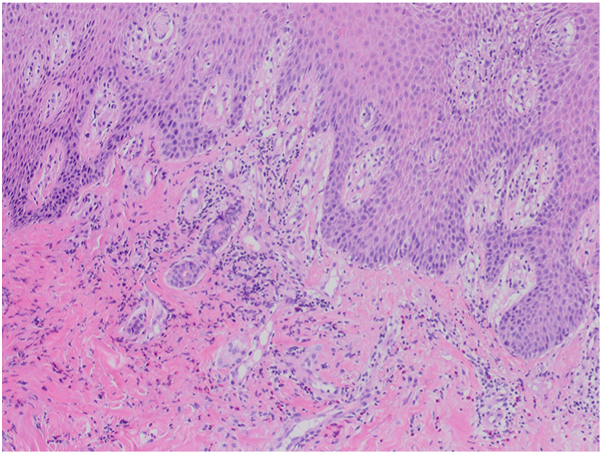
Histopathological examination of the biopsy specimen taken from the palm revealing hyperparakeratosis and perivascular lymphocytic infiltration consistent with palmoplantar pustular psoriasis (10×, H&E).

**Fig 6:**
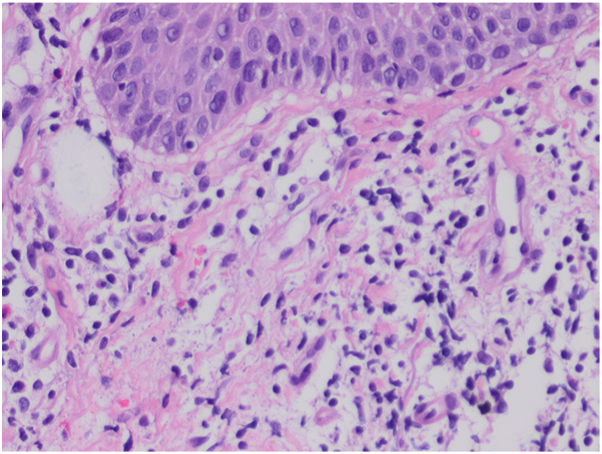
Higher magnification showing perivascular lymphocytic infiltration (40×, H&E).

**Fig 7:**
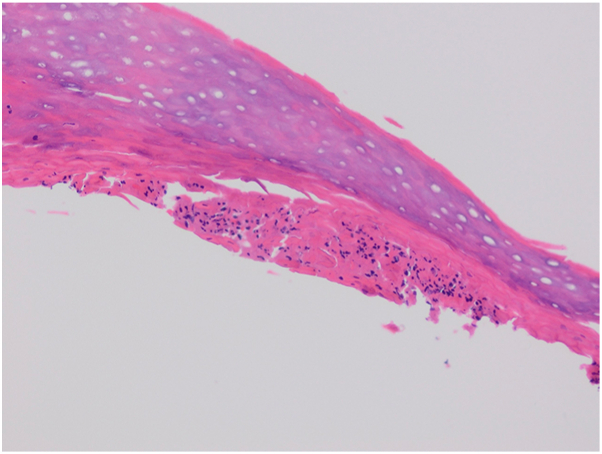
Histopathology showing a subcorneal pustule filled with neutrophils, consistent with the diagnosis of palmoplantar pustular psoriasis (20×, H&E).

A 62-year old male patient with a 30 year history of plaque psoriasis involving the extensor surfaces of the arms and legs, the back, and the external auditory canal and a psoriasis area and severity index score of 18 did not improve after courses of methotrexate, acitretin, and narrow-band ultraviolet B. He was started on the IL-17 A/F antagonist bimekizumab. During treatment with bimekizumab his initial plaque psoriasis lesions showed significant improvement. However following the third dose of bimekizumab, administered at week 8 according to the standard dosing regimen, he developed redness, scaling, and widespread pustules on the palms and soles (Figs 1 and 2).The patient’s biological therapy was stopped on the same day. A biopsy was taken from the pustules on the palm of the patient’s hand for histopathological examination to confirm the diagnosis. Considering the patient’s age and routine test results, treatment with 25 mg/d of acitretin and topical betamethasone valerate/fusidic acid was initiated. Within 14 days, there was a significant decrease in the number of new pustules, as well as improvement in erythema and scaling (Figs 3 and 4). Histopathological examination of the sections revealed hyperparakeratosis, spongiosis, subcorneal pustule formation, and lymphocytic infiltration, including a few neutrophils, around dilated capillary vessels in the papillary dermis (Fig 5, Fig 6, Fig 7).

## Discussion

The “cytokine shift” hypothesis is the most commonly accepted explanation for PR linked to biological therapy. This model suggests that effective suppression of the pathway being targeted leads to a shift in the immune response, activating alternative cytokine networks.^6^^,^^7^

Although the complex immunopathogenesis of PPP has not yet been fully defined, current evidence indicates that the Th17 pathway and the IL-17 cytokine play critical roles in its pathogenesis. IL-17 initiates an inflammatory reaction in diverse cell types such as keratinocytes, endothelial cells, chondrocytes, fibroblasts, and monocytes.^8^

Bimekizumab is the first approved biologic agent that blocks both IL-17A and IL-17F with high affinity.^5^ Dual blockade may result in deeper IL-17 suppression compared to IL-17A inhibitors alone. Consequently, there is a notable “rebound” activation of the type I interferon response, especially interferon-α, which is usually under the negative control of IL-17. This increase initiates an inflammatory cycle that leads to keratinocyte activation, neutrophil chemotaxis, and epidermal pustule formation.^8^, ^9^, ^10^, ^11^, ^12^

Paradoxical psoriasis reactions associated with anti-TNF medications, particularly occur in patients with those conditions like rheumatoid arthritis, inflammatory bowel disease. While anti-IL-17 drugs are not usually prescribed for rheumatoid arthritis or inflammatory bowel disease, these PR are most often seen in psoriasis patients.^9^^,^^10^^,^^13^

Anti-TNF agents tend to cause paradoxical psoriasiform reactions, typically within a year, while IL-17 inhibitors usually cause them within 6 months.^9^^,^^13^ Also, in paradoxical psoriasis from IL-17, the lesion form usually becomes pustule instead of guttate, unlike TNF-α inhibitors.^11^ In our case, PPP development began immediately after the third bimekizumab dose, which was administered in the eighth week, and it showed typical clinical and histopathological features. Although we cannot prove cause and effect, the absence of a previous history of pustular psoriasis, the rapid regression of lesions after discontinuing the drug, and the absence of other triggering factors provide strong evidence that this condition resulted from the drug.

To date, PR associated with bimekizumab have been rarely reported. The fact that bimekizumab is a relatively new drug may explain why there are limited reports of PR yet. Recently, Martora et al described a case of severe paradoxical scalp psoriasis induced by Bimekizumab.^14^ However our report describes a case of paradoxical palmoplantar pustular psoriasis associated with this agent. Our case suggests that the “cytokine shift” hypothesis may also apply to this new, potent agent. Future studies should address whether bimekizumab's simultaneous and potent blockade of both IL-17A and IL-17F leads to deeper IL-17 suppression and consequently a more pronounced rebound interferon-α effect compared to agents targeting only IL-17A.

In conclusion, we present a case of paradoxical PPP associated with bimekizumab treatment. We do not yet have a complete understanding of the long-term safety of new biological drugs like bimekizumab. Sharing clinical observations is crucial for identifying and addressing possible unexpected reactions.

## Conflicts of interest

None disclosed.

## References

[1] Alpsoy E., Polat M., Fettahlioglu-Karaman B. (2017). Internalized stigma in psoriasis: a multicenter study. J Dermatol.

[2] Bataille P., Layese R., Claudepierre P. (2022). Paradoxical reactions and biologic agents: a French cohort study of 9303 patients. Br J Dermatol.

[3] Shmidt E., Wetter D.A., Ferguson S.B., Pittelkow M.R. (2012). Psoriasis and palmoplantar pustulosis associated with tumor necrosis factor-α inhibitors: the Mayo clinic experience, 1998 to 2010. J Am Acad Dermatol.

[4] Gürsel Ürün Y., Yelgen H., Ürün M. (2022). Secukinumab-induced paradoxical palmoplantar pustular psoriasis. Turkderm.

[5] Ali Z., Matthews R., Al-Janabi A., Warren R.B. (2021). Bimekizumab: a dual IL-17A and IL-17F inhibitor for the treatment of psoriasis and psoriatic arthritis. Expert Rev Clin Immunol.

[6] Al-Homood I., Alaboon N., Draz H.E. (2025). Secukinumab-induced severe palmoplantar psoriasis treated with Guselkumab: a case report. Mediterr J Rheumatol.

[7] Ren J., Deng L., Guo S., Liu H. (2024). Paradoxical reaction to IL-17A inhibitor: a case report and literature review. Front Med (Lausanne).

[8] Frieder J., Kivelevitch D., Menter A. (2018). Secukinumab: a review of the anti-IL-17A biologic for the treatment of psoriasis. Ther Adv Chronic Dis.

[9] Singla S., Luz D. (2024). Paradoxical psoriasis with IL-17 inhibitors. Rheumatol Adv Pract.

[10] Xia P., Li Y.H., Liu Z. (2021). Recalcitrant paradoxical pustular psoriasis induced by infliximab: two case reports. World J Clin Cases.

[11] Wang Y., Yang F., Wang R., Luo S. (2024). Paradoxical psoriasis induced by IL-17 antagonists. Indian J Dermatol Venereol Leprol.

[12] Lu J., Lu Y. (2023). Paradoxical psoriasis: the flip side of idiopathic psoriasis or an autocephalous reversible drug reaction?. J Transl Autoimmun.

[13] Brown G., Wang E., Leon A. (2017). Tumor necrosis factor-α inhibitor-induced psoriasis: systematic review of clinical features, histopathological findings, and management experience. J Am Acad Dermatol.

[14] Martora F., Battista T., Potestio L., Megna M. (2025). Severe paradoxical scalp psoriasis induced by Bimekizumab in a young multifailure Hidradenitis Suppurativa patient. Dermatol Ther (Heidelb).

